# Supplementary material for: Performance of prototype serological immunoassays for foot-and-mouth disease virus using G-H loop peptides and stabilized virus-like particles
Source: Microbiol Spectr. 2026 Apr 27;14(6):e03514-25. doi: 10.1128/spectrum.03514-25 (PMC13227964; doi:10.1128/spectrum.03514-25)
Supplement: Figure S1 and Table S1 — ROC analysis of sera reactivity and a list of tested bovine sera, [file spectrum.03514-25-s0001.docx]

**Figure S1**: ROC analysis of sera reactivity against peptides and VLPs in different dilutions for each serotype O KEN/4/2018, A SUD/9/2018, SAT1 TAN/22/2014 and SAT2 KEN/19/2017.

Table S1: Tested bovine sera samples against the four serotypes.

| Sample ID | Isolate | Lineage | (infected or/and vaccinated) |
| --- | --- | --- | --- |
| 22 | O ETH/38/2005 | EA-3 | N/A |
| 104626 | O UGA/03/2002 | EA-2 | 21d |
| 404622 | O UGA/03/2002 | EA-2 | 21d |
| 304628 | O UGA/03/2002 | EA-2 | 21d |
| 304049 | O KEN/77/1978 | EA-1 | 21d |
| 404650 | O KEN/77/1978 | EA-1 | 21d |
| 404050 | O KEN/77/1978 | EA-1 | 21d |
| c21-1817 | A ERI 1998 | G-IV | Vaccinated |
| RZ71 | A ERI/3/1998 | G-IV | Vaccinated |
| RZ73 | A ERI/3/1998 | G-IV | Vaccinated |
| 602779 | A ETH/4/2007 | G-VII | N/A |
| 302763 | A ETH/4/2007 | G-VII | N/A |
| 24 | A ETH/7/2008 | G-VII | N/A |
| 10 | A ETH/6/2000 | G-VII | N/A |
| ANIMAL 21129 | SAT1 RHO/12/1978 | II | Vaccinated |
| VP 80 | SAT1 RHO/12/1978 | II | Vaccinated |
| VP 81 | SAT1 RHO/12/1978 | II | Vaccinated |
| VP 83 | SAT1 RHO/12/1978 | II | Vaccinated |
| VP 84 | SAT1 RHO/12/1978 | II | Vaccinated |
| 5518 | SAT2 ZIM/5/1987 | II | Infected and vaccinated |
| 8812 | SAT2 ZIM 5/1987 | II | Infected and vaccinated |
| 403739 | SAT2 ETH/65/2009 | XIII | Vaccinated |
| 404077 | SAT2 ETH65/2009 | XIII | Vaccinated |
| FMD287 | SAT2 ERI | VII | Vaccinated |
| VL 80 | SAT2 ERI | VII | Infected and vaccinated |
| VL 92 | SAT2 ERI | VII | Infected and vaccinated |
